# Longitudinal, Multimodal Tracking Reveals Lasting Neurovascular Impact of Individual Microinfarcts

**DOI:** 10.1002/advs.202417003

**Published:** 2025-03-31

**Authors:** Yifu Jin, Fei He, Haad Rathore, Yingchu Sun, Jia‐ao Zhang, Xinyu Li, Rongkang Yin, Hanlin Zhu, Chong Xie, Lan Luan

**Affiliations:** ^1^ Department of Electrical and Computer Engineering Rice University Houston TX 77005 USA; ^2^ Neuroengineering Initiative Rice University Houston TX 77005 USA; ^3^ Applied Physics Program Rice University Houston TX 77005 USA; ^4^ Department of Bioengineering Rice University Houston TX 77005 USA

**Keywords:** electrophysiology, flexible electronics, microinfarcts, neural activity, neuroimaging, two‐photon imaging

## Abstract

Microinfarcts, the “invisible lesions”, are prevalent in aged and injured brains and associated with cognitive impairments, yet their neurophysiological impact remains largely unknown. Using a multimodal chronic neural platform that combines functional microvasculature imaging with spatially resolved neural recording, the neurovascular effect of a single microinfarct is investigated. Unlike larger strokes, microinfarcts induced only temporary suppression of neural activity with minimal cell death, with recovery paralleling vasculature remodeling at the infarct core. Neural activity is more severely suppressed at the shallower cortical layer despite milder vascular damage compared to deeper layers, and the excitability of fast‐spiking interneurons attenuation is accompanied by heightened bursting of regular spiking neurons. Spike phase locking at the low‐gamma band is disrupted, indicating a lasting impairment of long‐range assembly communication. These results highlight the subtle yet significant neurovascular disruptions of a single microinfarct.

## Graphical abstract

Jin et al. investigate the neurovascular impact of microinfarcts, revealing transient neural suppression with minimal cell death and recovery linked to vascular remodeling. Fast‐spiking interneurons exhibit reduced excitability, whereas regular‐spiking neurons showed increased bursting activity. Disruptions in low‐gamma phase locking suggest persistent network instability. These findings underscore the subtle, yet lasting impairments caused by a single microinfarct.

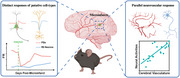

## 1 Introduction

Microinfarcts, microscopic ischemic injuries with tissue necrosis size ranging from 0.2 to 2.9 mm in diameter, are frequently found in aging brains but are often too small to be resolved with clinical imaging techniques.^[^ ^1^, ^2^, ^3^ ^]^ They often co‐occur with numerous pathological conditions, including cerebrovascular diseases such as macro‐infarcts and intracerebral hemorrhages,^[^ ^4^ ^]^ advanced small vessel diseases such as arteriolosclerosis^[^ ^5^ ^]^ and cerebral amyloid angiopathy,^[^ ^6^, ^7^ ^]^ and neurodegenerative conditions such as Alzheimer's disease,^[^ ^8^ ^]^ Parkinson's disease,^[^ ^9^ ^]^ and Lewy body diseases.^[^ ^10^ ^]^ Despite their prevalence, the neurophysiological consequences of microinfarcts largely remain an enigma.

The current understanding of microinfarcts mainly stems from neuropathological examination from standard brain autopsy^[^ ^11^, ^12^ ^]^ and high‐resolution MRI sequences.^[^ ^12^, ^13^, ^14^ ^]^ Techniques such as 7 T structural MRI^[^ ^12^ ^]^ and diffusion‐weighted imaging^[^ ^13^, ^14^ ^]^ have enabled the detection of microinfarcts, but they have limited detection time windows and resolution to resolve pathophysiological changes in the perilesional tissue.^[^ ^15^, ^16^ ^]^ Neuropathological studies primarily focus on chronic cerebral microinfarcts in association with other pathologies,^[^ ^17^ ^]^ overlooking the temporal dynamics and the isolated impact of microinfarct itself.

Mouse models of cerebral microinfarcts show remarkable similarity to human cerebral microinfarcts with respect to their range of shapes, size, and location, the observed functional deficits, and their limited detectability in clinical imaging modalities.^[^ ^1^, ^2^, ^3^ ^]^ Various methodologies have been developed to induce microinfarcts in mouse models, including injecting microbeads to occlude numerous capillaries^[^ ^18^, ^19^ ^]^ and single‐vessel‐targeted photothrombosis that occludes individual penetrating arterioles and venules,^[^ ^20^, ^21^, ^22^ ^]^ which creates a small lesion with a size <1 mm in diameter.^[^ ^23^ ^]^ Studies leveraging high‐resolution imaging techniques and behavioral assessments reported persistent hemodynamic deficits and noticeable behavioral impairments following microinfarcts; yet, the lack of longitudinal neural measurement prevents the understanding of how these deficits evolve post‐microinfarct.^[^ ^22^ ^]^ However, direct measurements of the neural outcome of microinfarcts are scarce, with one of the few studies reporting an absence of alteration in functional connectivity from mesoscopic imaging, presenting a striking discrepancy with persistent behavioral impairments.^[^ ^24^ ^]^ The neural impact of microinfarcts and their association with vascular dysfunctions remain unclear, mainly due to the difficulty of directly measuring and longitudinally tracking neural activity post‐microinfarcts at adequate resolutions, particularly while monitoring vascular and other activities simultaneously.

**Figure 1:**
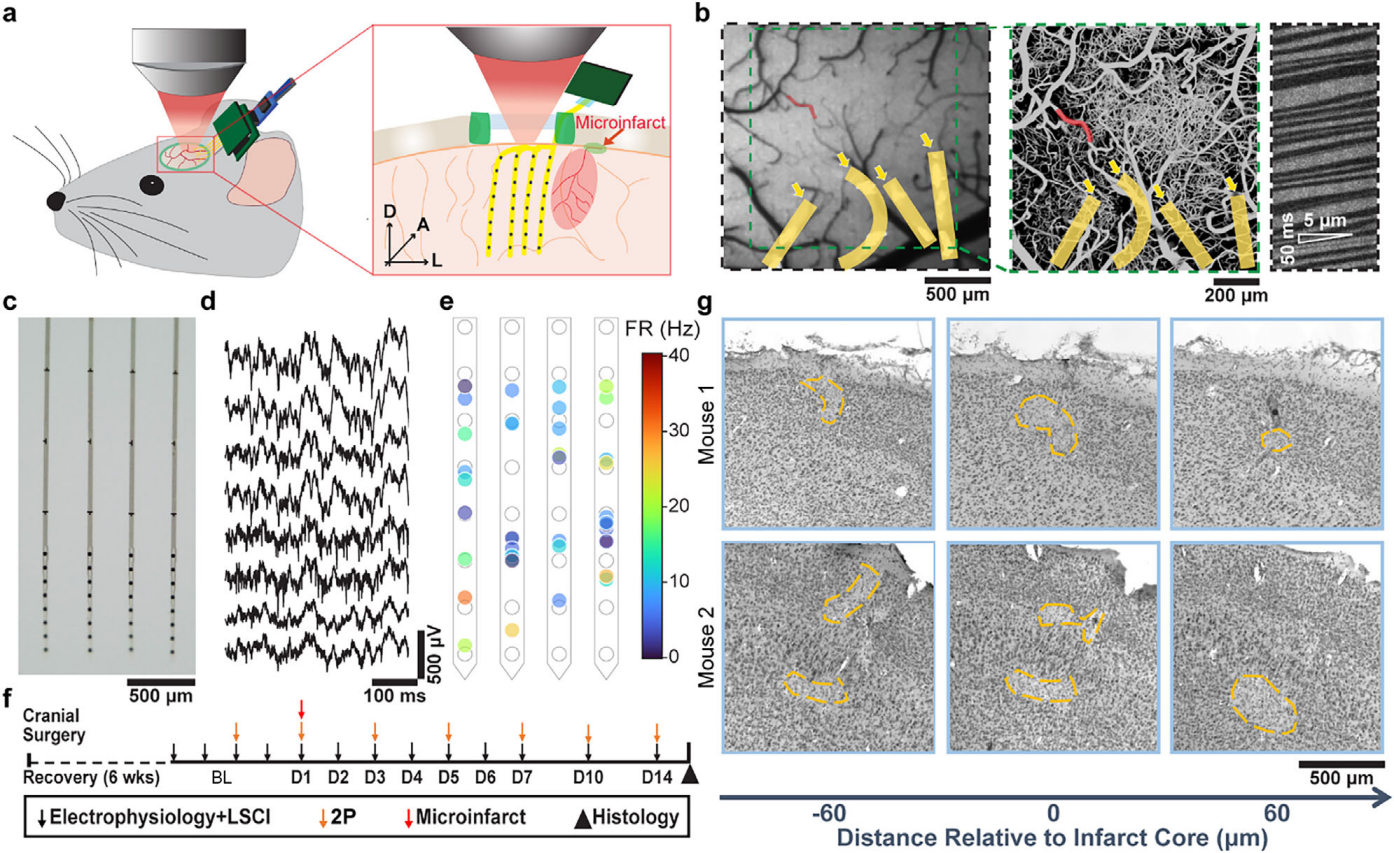
Multimodal, longitudinal tracking of neural and microvascular responses to microinfarcts. a) Schematics showing closely integrated two‐photon (2P) imaging and multi‐shank, intracortical recording. The arrows indicate the anatomical orientation: lateral (L), anterior (A), and dorsal (D). b) Three imaging techniques bridging mesoscopic and microscopic resolutions: Laser speckle contrast imaging (LSCI) of cerebral blood flow (CBF, left), 2P z‐stack imaging of microvasculature (middle), and 2P line‐scans (2PLS) along a segment of a capillary to infer red‐blood cell velocity (right). The red curve marks the vessel segment for targeted photothrombosis. The capillary of 2PLS is not marked. Nanoelectronic threads (NETs) are located by the autofluorescence of their gold interconnects and pseudo‐colored as golden ribbons. c) Photo of a 4‐shank NET in water. d) Representative raw recording traces from a single shank. e) Representative recording showing the firing rate and estimated location of the units projected onto the recording sites (white circles). f) Experimental timeline. “BL” refers to the pre‐microinfarct baseline. g) Nissl staining of 30 µm coronal sections showing scattered neuronal death at the microinfarct core (highlighted with yellow polygons).

In this study, we longitudinally tracked neural activity, cerebral blood flow (CBF), and microvasculature remodeling after the induction of microinfarcts in the same brain region. These multimodal measurements were enabled by integrating intracortical electrophysiological recording with various optical methods for single‐vessel‐targeted photothrombosis and imaging of microvasculature and microcirculation (**Figure** **1**a,b). We used ultraflexible polymer electrodes, the nanoelectronic threads (NETs),^[^ ^25^ ^]^ for intracortical laminar recording (Figure 1c–e). NETs form an intact tissue‐electrode interface, enabling the investigation of neurovascular activity at minimal disruption to the baseline microvasculature and neurophysiology.^[^ ^26^ ^]^ NETs are also compatible with chronic optical imaging in the same region,^[^ ^25^, ^26^, ^27^ ^]^ allowing for high‐resolution, cortical‐depth‐specific measurements of neurovascular responses to microinfarcts over time. We use healthy aged mouse models because they more accurately reflect the vascular, inflammatory, and neurodegenerative changes associated with aging while avoiding confounding factors from comorbid diseases that often coexist with microinfarcts in humans. We aim to determine the spatial extent and temporal progression of both neural and vascular impairments, thus uncovering the neurovascular impact of a single microinfarct.

## 2 Results

Unraveling the neurovascular impact of microinfarcts demands high‐resolution mapping of neural activity and microcirculation longitudinally following the formation of these microscopic lesions. To meet these demands, we co‐implanted 4‐shank NETs and a cranial window over the primary motor cortex.^[^ ^28^ ^]^ This enabled simultaneous recording of laminar neural activity and imaging of the microvasculature and cerebral blood. To minimize optical obstruction, we specifically designed NETs with a thin shank width of 50 µm, reducing imaging interference to a level comparable to that of a surface vessel. We performed imaging and recording sequentially on the same day, eliminating any concerns about the photovoltaic effect.

Experiments began 6 weeks post‐surgery to allow vascular and neuronal remodeling induced by the implantation of NETs and the cranial window to subside.^[^ ^25^, ^26^ ^]^ We induced single‐vessel targeted photothrombotic occlusion by patterned illumination with a digital micromirror device (DMD).^[^ ^29^ ^]^ Longitudinal, multimodal measurements of neurovascular activity started from pre‐microinfarct baseline (BL) and continued to two weeks post microinfarct, a period determined from our pilot experiments as the time when neurovascular activity had restored and became relatively stable, followed by terminal histology (Figure 1f). Post‐mortem histology confirmed the formation of microscopic ischemic lesions with scattered, limited neuronal loss (Figure 1g). To avoid confounds from anesthesia,^[^ ^30^, ^31^ ^]^ we conducted these multimodal measurements on awake, head‐fixed animals.

### 2.1 Transient Impairment of Microvasculature and Microcirculation Following a Microinfarct

**Figure 2:**
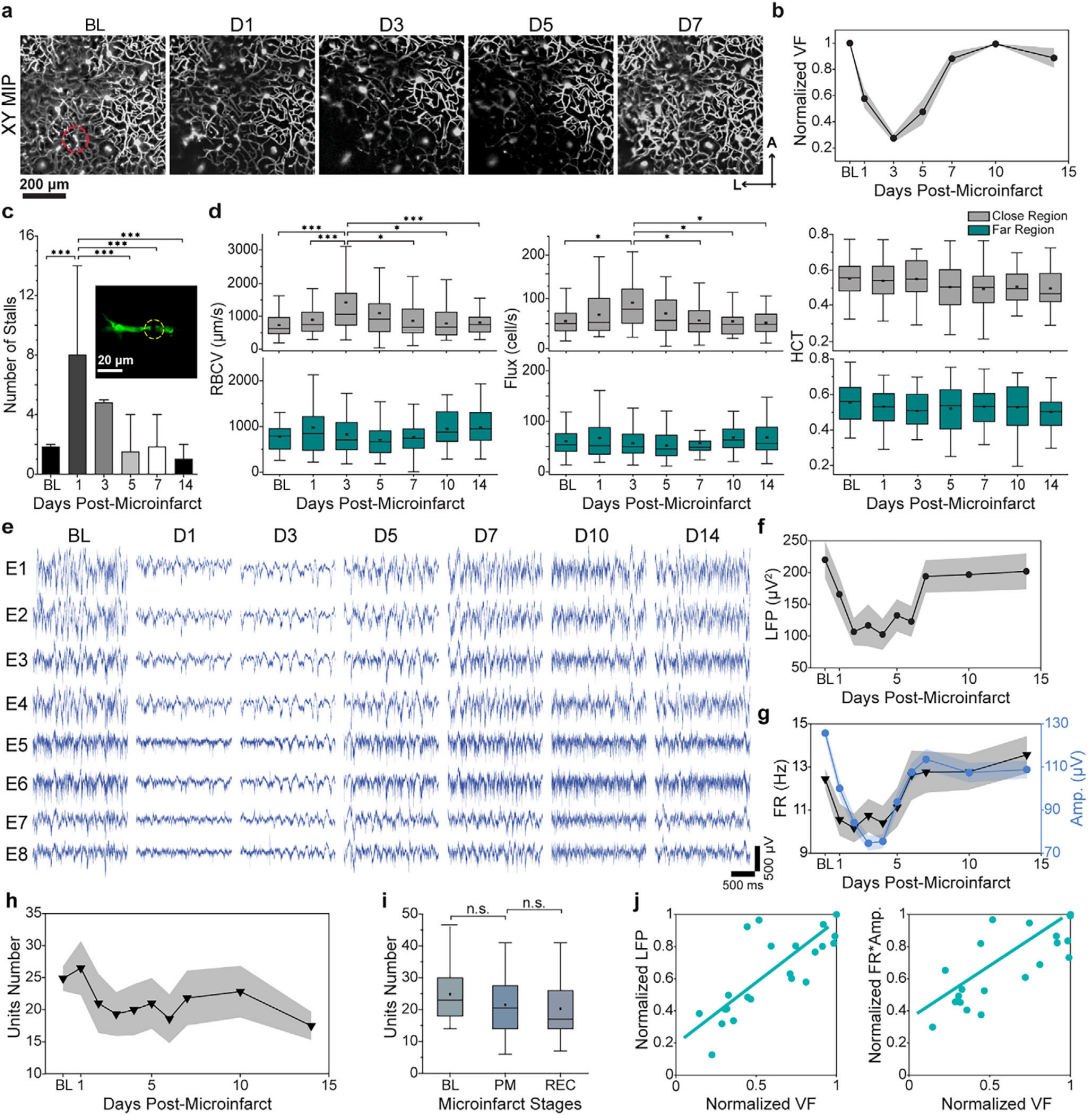
Impairment of peri‐lesional neural activity paralleled capillary volume fraction at the microinfarct core. a) Representative maximum intensity projection (MIP) images of microvasculature (250–350 µm from brain surface). Arrows indicate the anatomic direction of lateral and anterior. b) Normalized capillary volume fraction over time. Shade indicates ±SE (n = 6 mice). c) Bar graphs of capillary stall numbers over time (n = 6 mice). Error bars represent 1.5 times the interquartile range (IQR). d) Box plots of red blood cell velocity (RBCV, left), flux (middle), and HCT (right) over time. The central box spans 25th–75th percentiles, with the line representing the median and the square marking the mean value. Whiskers show minimum and maximum values within 1.5 times the IQR. e) Representative raw recording traces from one location across cortical depths over time relative to microinfarct. f,g) Average spectral power of local field potential (LFP) at 30–60 Hz (n = 22 shanks from 6 mice), spike firing rate, and spike amplitude for all sorted units from 6 mice over time. Shade indicates ± SE. h) Average unit numbers from 6 mice over time; shade indicates ± SE. i) Box plots of unit numbers for the BL, post‐microinfarct (PM), and recovery (REC) phases. j) Scatter plots of normalized capillary volume fraction at the infarct site versus peri‐lesional LFP (left) and versus firing rate × amplitude (right) across all recording locations and time points, showing linear correlation. Pearson's correlation coefficients: ρ = 0.84, *P* < 0.0001 (left); ρ = 0.68, *P*<0.0001 (right). Significance levels: n.s., no significance; ^*^ *P* < 0.05; ^**^ *P* < 0.01; ^***^ *P* < 0.001.

To elucidate the duration and spatial extent of vascular impairment induced by a single microinfarct, we performed longitudinal two‐photon (2P) imaging of microvasculature and microcirculation. In the days immediately following the induction of microinfarcts, we observed worsening vascular damage around the microinfarct in the initial days, marked by an expansion of non‐perfused microvasculature volume. Subsequently, vascular remodeling and angiogenesis occurred, leading to increased microvasculature density in the following days (**Figure** **2**a). The volume fraction of microvasculature surrounding the microinfarct core reached the lowest level at Day 3 (72 h) post‐infarct induction. It gradually recovered to BL levels by Day 7 and remained stable thereafter (Figure 2b).

In addition to direct damage to the vasculature, microinfarcts can also compromise local perfusion by affecting microcirculation in structurally intact vasculature.^[^ ^32^ ^]^ This often involves capillary stalling (Figure 2c), the temporary arrest of capillary blood flow typically caused by a stalled leukocyte^[^ ^33^ ^]^ or constricted pericytes.^[^ ^34^ ^]^ We, therefore, examined the changes in capillary stall numbers over time following the microinfarct in regions within 600 µm from the microinfarct core. Stall numbers surged on the day of induction, then declined by Day 3 and stabilized at BL levels. Using line scans along individual capillary segments,^[^ ^35^ ^]^ we quantified red blood cell velocity (RBCV), flux, and the hematocrit ratio (HCT). RBCV and flux in the region adjacent to the infarct core (<250 µm) significantly increased on Day 3 post microinfarct and returned to BL levels by Day 7, likely as a compensatory response to the vasculature damage at the core and the stalled capillary in the nearby region. In contrast, the RBCV and flux in the region farther away from the infarct core (>250 µm), along with HCT at all locations, showed no significant difference across time (Figure 2d). These findings suggest that microinfarcts result in a brief period of microvasculature damage, accompanied by transient abnormalities in the microcirculation of the surrounding tissue.

### 2.2 Parallel Dynamics of Peri‐Lesional Neural Activity and Microvasculature

We distributed ultraflexible NETs in the peri‐lesional regions at distances of 0.1–1.1 mm to the microinfarct core (Figure S1a, Supporting Information), targeting close‐by areas where microvasculature was largely unaffected by the microinfarct. Broadband intracortical neural activity in these regions followed a similar time course of suppression and recovery as the microvasculature in the core. The neural activity showed the most pronounced suppression between Day 2–4, followed by a gradual return to the BL level by Day 7–10 (Figure 2e). Quantification of 30–60 Hz local field potential (LFP) spectral power (Figure 2f), spiking rate of single and multi‐units, and spike amplitudes (Figure 2g), revealed a consistent longitudinal pattern. Unlike larger strokes,^[^ ^36^ ^]^ we observed no distance dependency in the time course of neural recovery within the measured range: regions closer to the microinfarct took a similar duration to recover as those farther away despite experiencing more substantial initial suppression (Figure S1b, Supporting Information). Consequently, we did not differentiate recording locations in the time course analysis. We defined Days 1–6 as the post‐microinfarct (PM) period, characterized by significant neural and vascular deficits, and Days 7–14 as the recovery (REC) period, during which neurovascular deficits subsided. We observed that the spectral power of higher‐frequency oscillations (60–110 Hz) exhibited a similar recovery pattern (Figure S1d–g, Supporting Information), whereas lower‐frequency oscillations (0.5–4 Hz, 4–8 Hz, 8–12 Hz, 12–30 Hz) remained relatively preserved and were less affected by the microinfarct (Figure S1h,i, Supporting Information). The resilience of lower‐frequency oscillations may be attributed to their strong dependence on widespread network activity and subcortical inputs,^[^ ^37^, ^38^ ^]^ which remain relatively intact despite localized cortical damage. Additionally, the number of recorded units, including single and multi‐units, remained relatively stable, indicating minimal changes in the population of active neurons after microinfarcts (Figure 2h,i).

Given that the neural population had little reduction following the microinfarct, we used LFP and spiking rate × amplitudes (FR × Amp) to quantify neural impairment and recovery, which represent collective activity from surrounding tissue and localized individual neuron activity, respectively. Notably, these values closely tracked the volume fraction of microvasculature throughout the measurements (Figure 2j). Across all time points, locations, and animals, Pearson's correlation coefficients were ρ = 0.84, p < 0.001 for BL normalized LFP and vasculature volume fraction (Figure 2j, left), and ρ = 0.68, p < 0.001 (Figure 2j, right) for FR × Amp and vasculature volume fraction. Despite the difference in correlation coefficients, both low‐frequency LFP and high‐frequency unit activity exhibited significant positive correlations, highlighting the consistent relationship between peri‐lesional activity impairment and capillary volume reduction in the microinfarct core. Neural activity suppression was observed across all recording sites, suggesting that microinfarcts impact neural activity beyond the regions with direct microvasculature impairment and extended at least 1 mm away. Furthermore, the time course and magnitude of neural impairment in the peri‐lesional region with intact vasculature closely mirrored the disruption and recovery of microvasculature and perfusion at the infarct core.

### 2.3 Differential Depth Dependence of Neural and Vascular Impairment

Was the neural impairment caused by microinfarcts correlate with microvascular impairment at a finer resolution? To answer this question, we utilized the depth resolution of two‐photon imaging and laminar intracortical recording to examine the cortical depth profile of spiking rates and microvasculature volume density, given that observed changes in neuronal populations and microcirculation were minimal or mild throughout the measurement period.

**Figure 3:**
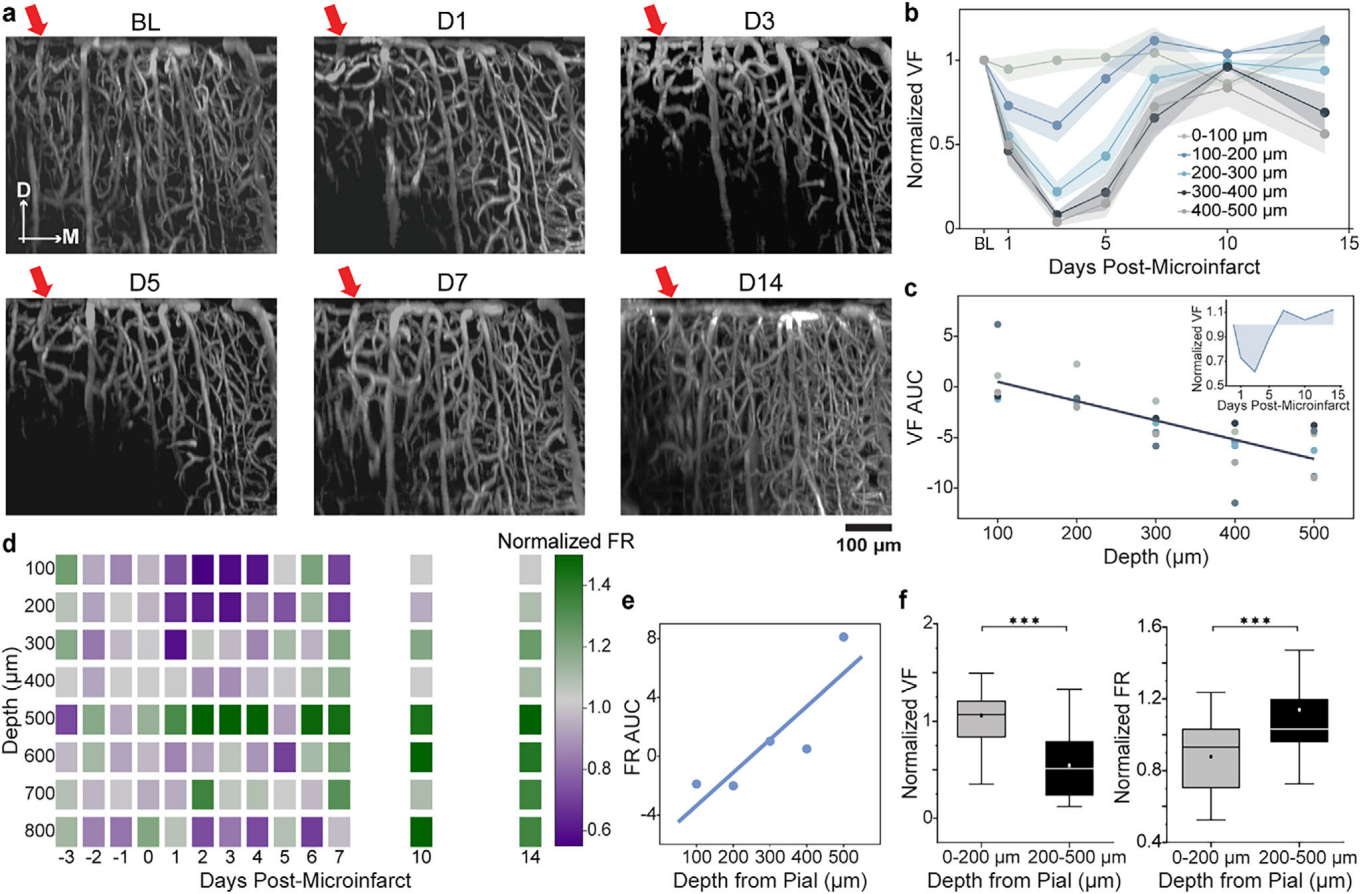
Distinct cortical depth dependence of neural and vascular response to microinfarct. a) Representative YZ‐plane MIP from 2P vasculature imaging, showing more severe loss of microvessels in deeper cortical regions. Red arrows indicate the targeted penetrating arteriole. White arrows indicate the medial (M) and dorsal anatomic direction. b) Depth‐specific volume fraction of vasculature as a function of days following microinfarct. Shade indicates ± SE (n = 6). c) AUC of vasculature volume fraction (VF) as a function of depth from the pia layer. VF was calculated for every 100 µm depth interval, with the lowest depth of each section indicated on the graph. Inset shows an example of AUC as the shaded area. Pearson's correlation coefficient: ρ = −0.978, *P* = 0.0038. d) Depth‐specific single‐unit firing rate normalized to the BL (n = 5). e) Firing rate AUC post‐microinfarct as a function of depth from the pia layer. Pearson's correlation coefficient: ρ = 0.892, *P* = 0.04. f) Box plots showing statistical significance in the cortical depth difference in the vasculature volume fraction (left) of 6 mice and single‐unit firing rate of 5 mice post‐microinfarct (right). Percentiles, median and mean values, and IQR are depicted in Figure 2i. Significance levels: n.s., no significance; ^*^ *P* < 0.05; ^**^ *P* < 0.01; ^***^ *P* < 0.001.

We sectioned the z‐stack imaging of microvasculature along the YZ plane to reveal the depth profile of vascular damage. Following the occlusion of a single penetrating arteriole, the vasculature within 100 µm depth from the surface remained largely unaffected. In contrast, deeper layers showed more severe and persistent vasculature damage, with a longer duration and broader spatial impact than shallower layers (**Figure** **3**a,b). The reduced damage to near‐surface microvasculature can be attributed to the cortical depth distribution of microvasculature branching from penetrating arterioles.^[^ ^39^ ^]^ Penetrating arterioles have fewer side branches in superficial layers, with side branch density peaking at a cortical depth of 500–600 µm. Moreover, photothrombotic occlusion of penetrating arterioles consistently reduced the blood flow in the first 3–4 branches, regardless of depth,^[^ ^39^ ^]^ leading to larger non‐perfused areas in deeper regions compared to superficial layers. To quantify the combined effect of damage duration and severity, we calculated the “area under the curve (AUC)” of the vasculature volume fraction normalized to BL (Figure 3c). In this analysis, negative AUC values indicate a reduction in volume fraction, while zero represents no longitudinal changes. Notably, the AUC was close to zero in the shallowest 100 µm and progressively decreased with increasing depth, reflecting more severe vascular impairment in deeper layers.

In contrast, neural activity displayed a different depth‐dependent pattern. Although fluctuations in the averaged single‐unit firing rate were observed at each depth, attenuation was more pronounced and sustained in the shallow layers. Interestingly, layer 4 (at a depth of 500 µm) showed a notable increase in firing rate, rather than the expected attenuation, following the microinfarct (Figure 3d). Consistently, the AUC of firing rate over time, using the same sign convention as Figure 3c, showed a reverse depth dependence (Figure 3e), with the most significant reduction occurring within 200 µm of the cortical surface. The difference between shallow and deep layers in the volume fraction of vasculature and neural activity was statistically significant (p < 0.001, Figure 3f). The disparity in the depth‐dependence of neural and vascular deficits suggests that the attenuation of neural activity in shallow layers is not solely a result of reduced metabolic support from compromised vasculature in the surrounding area.

### 2.4 Attenuation of FSIs Accompanied by Heightened Bursting of RS Cells Following Microinfarcts

**Figure 4:**
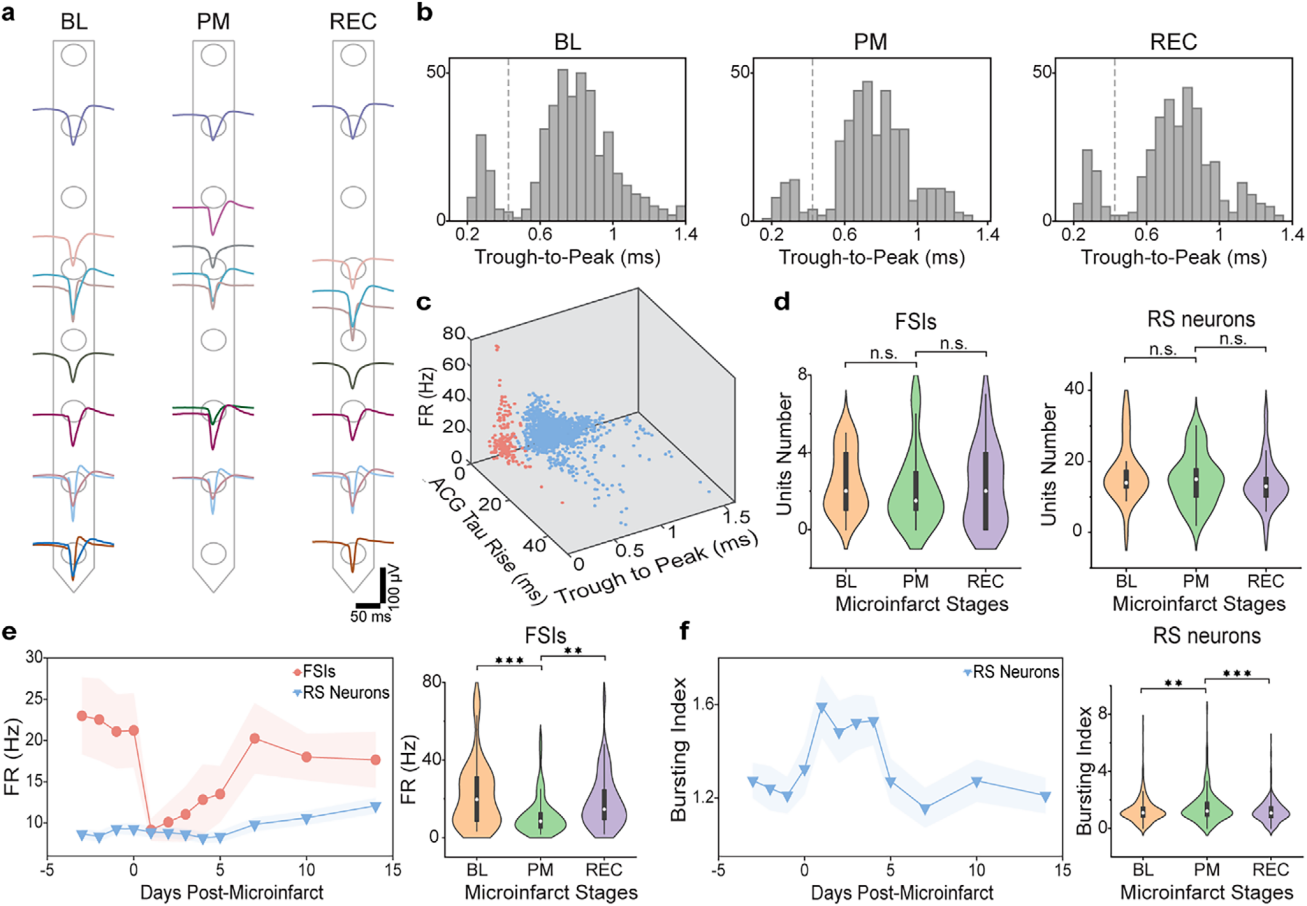
Attenuation of fast‐spiking interneurons (FSIs) and increased bursting of regular spiking (RS) cells following microinfarct. a) Example recordings of single units from a single NET shank at three stages of the microinfarct. Circles denote the recording site. Waveforms of recorded units are placed on the estimated coordinates based on triangulation. b) Distribution of trough‐to‐peak values across three stages of microinfarct showing consistency. Dashed lines mark 0.425 ms, the threshold for separating two putative cell types. c) Scatter plot of all single units in the parameter space of trough‐to‐peak latency, auto‐correlogram (ACG) rise time, and firing rate. Colors represent putative classifications of FSIs (red) and RS neurons (blue). d) Violin plots showing no significant population changes in the two putative cell types. White dots denote the median; box denotes the 25th–75th IQR; whiskers mark the range of 1.5 times IQR. e,f) Left: average firing rate per unit of FSIs and RS neurons (e) and average bursting index of RS neurons (f) as a function of days following microinfarct for all sorted units from 6 mice. Shade indicates ± SE. Right: violin plots showing the significant changes across the three stages of mcroinfarct. Significance levels: n.s., no significance; ^*^ *P* < 0.05; ^**^ *P* < 0.01; ^***^ *P* < 0.001.

During chronic implantation, the ultraflexible NETs maintained tight integration with the tissue, consistently detecting units with similar waveforms at the same recording sites throughout the longitudinal experiment (**Figure** **4**a). Despite a significant reduction in spike amplitudes and firing frequency following the microinfarct, as depicted in Figure 2g, the trough‐to‐peak values remained stable throughout this period (Figure 4b). This allows us to conduct a waveform‐based classification^[^ ^40^ ^]^ of a total of 1352 recorded neurons across all sessions into two distinct clusters (Figure 4c): narrow waveform units (trough‐to‐peak ≤ 425 µs), primarily representing fast‐spiking interneurons (FSIs), and the remaining units categorized as regular spiking (RS) neurons, predominantly excitatory cells with broader spike waveforms. Notably, neither cell category exhibited significant changes in active cell numbers across the BL, PM, and REC phases (Figure 4d).

The activity pattern of these two categories of cells showed distinctive responses to microinfarcts. The averaged firing rate of RS neurons exhibited minimal change post‐microinfarct, while the averaged firing rate of FSIs declined by over 50% on the day of the microinfarct, followed by a gradual increase from Day 2 to Day 7, subsequently returning to the BL levels in the REC phase (Figure 4e). This temporal pattern closely mirrored the overall neural activity in Figure 2f,g, suggesting that the attenuation of the activity of FSIs primarily drove the reduction in overall neural activity. However, the decrease in the activity of FSIs did not correlate with an increase in the RS activity when measured by the average population rate.

To further explore the fine temporal dynamics, we examined neuronal bursting, a phenomenon marked by rapid spiking followed by periods of inactivity.^[^ ^41^ ^]^ We focused on bursting events of the RS neurons, as they are linked to excitotoxicity in various neurological and physiological conditions.^[^ ^4^ ^]^ The Royer bursting index,^[^ ^41^ ^]^ averaged from all RS neurons, remained stable during BL sessions, increased significantly during the PM phase, and then subsided (Figure 4f). The increase in bursting index on Days 1–4 was statistically significant compared to both the BL (p < 0.001) and the REC phases (p < 0.01). The timing and duration of this marked surge in RS neurons bursting aligned with the reduction in inhibitory activity, underscoring the association between abnormal neural dynamics and the attenuation of inhibitory control following microinfarcts.

### 2.5 Alteration of Spike‐Field Phase Locking and Population Coupling Post Microinfarct

**Figure 5:**
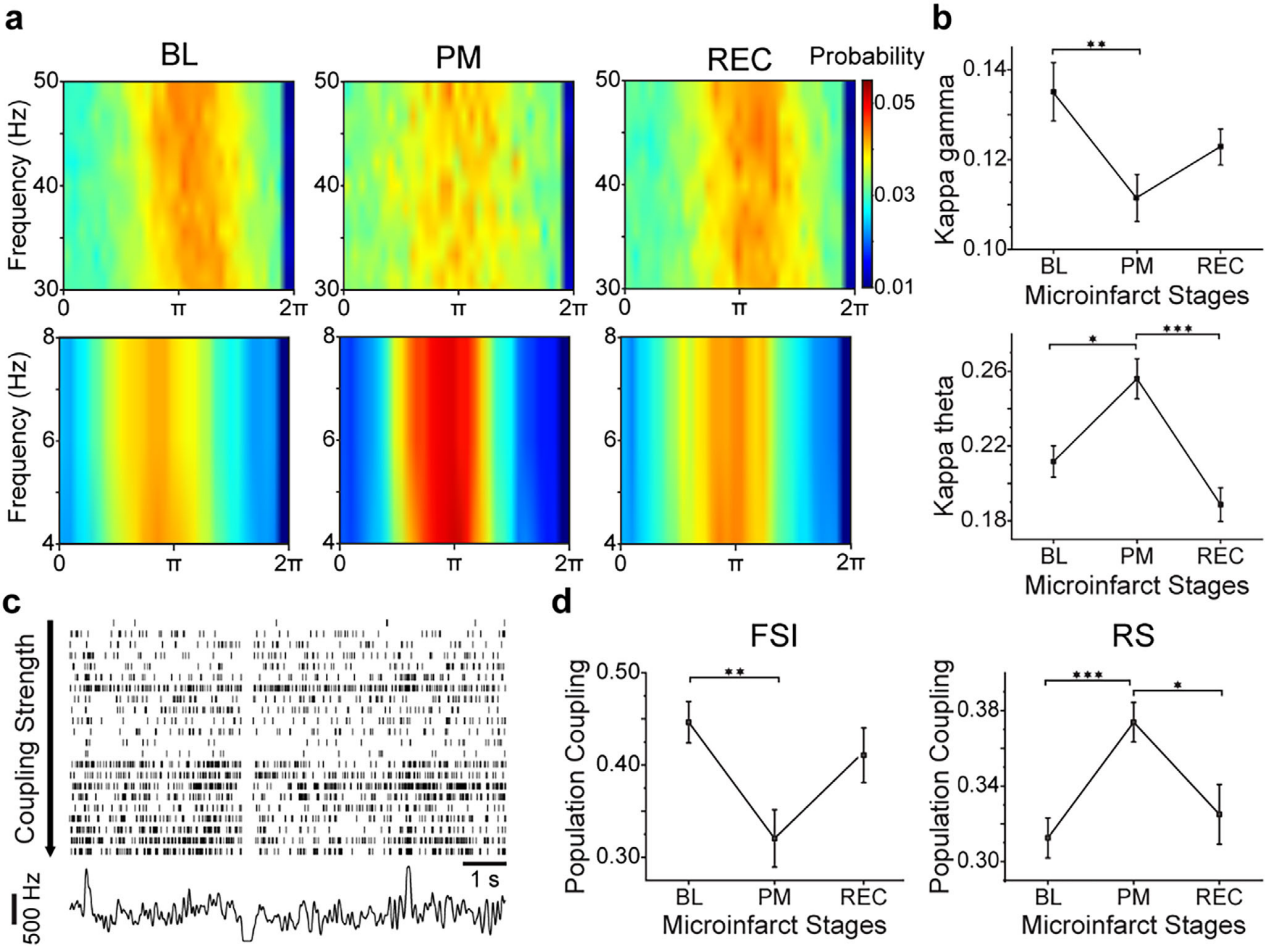
Alteration of spike‐field phase locking and population coupling postmicroinfarct. a) Gamma‐band (top row) and theta‐band (bottom row) spike phase histogram at three stages of microinfarct. b) Average modulation index Kappa of gamma band (top) and theta band (bottom) for all channels that passed permutation testing from 4 mice at three stages of microinfarcts. Error bars represent ± SE. c) Spike trains from a representative recording of 22 single units (arranged in descending order of coupling strength) and the population firing rate. d) Average population coupling coefficients of FSIs (left) and RS neurons (right) for all units from 6 mice at three stages following the microinfarcts. Error bars represent ± SE. Significance levels: n.s., no significance; ^*^ *P* < 0.05; ^**^ *P* < 0.01; ^***^ *P* < 0.001.

To further investigate changes in neuronal communication and functional connectivity following microinfarct, we computed the spike‐field phase locking^[^ ^42^ ^]^ at gamma frequency, due to its association with synchronous activity of FSIs, and at theta frequency, given theta rhythm's role in modulating gamma power over long distances^[^ ^43^ ^]^ and its link to motor planning and sensorimotor integration^[^ ^44^ ^]^ (**Figure** **5**a). Post‐microinfarct, spike phase locking to gamma frequency was significantly reduced, while the phase locking to theta frequency increased significantly (Figure 5b; Figure S4a, Supporting Information). Additionally, we assessed population coupling, defined as the synchronization of each neuron's spiking activity with the population activity within a 10 ms window (Figure 5c). Following the microinfarct, the population coupling of FSIs decreased significantly, whereas the coupling of RS neurons increased notably (Figure 5d; Figure S4b, Supporting Information). These changes indicate that reduced inhibitory coordination among FSIs weakened local synchrony and gamma coherence. Meanwhile, enhanced coupling among RS neurons and spike coherence in theta rhythm may reflect a compensatory response. Together, these alterations suggest a disruption of normal neural circuitry where reduced FSI coupling limits inhibitory regulation and potentially shifts the network toward excitatory‐dominated dynamics.

## 3 Discussion

Compared to extensive studies of ischemic strokes,^[^ ^45^, ^46^ ^]^ microinfarcts in aged populations are relatively understudied. While in vivo imaging and behavioral evaluation techniques have identified hemodynamic and behavioral deficits following microinfarcts using rodent models,^[^ ^22^, ^24^ ^]^ their direct neural impact and temporal evolution remain mostly unexplored. In this study, we employed a suite of advanced neurotechnology to investigate the longitudinal neurovascular impact of microinfarcts in aged mice at microscopic resolution. By combining multi‐site intracortical recordings across cortical depths with two‐photon imaging of microvasculature and microcirculation, we realized direct tracking and comparison of the time course, duration, and magnitude of the multifaceted changes induced by microinfarcts, offering a holistic depiction of the neurovascular impact of a single microinfarct—the smallest ischemic injury studied at this level of resolution.

Following a microinfarct, we detected that the suppression and subsequent recovery of neural activity in the peri‐infarct region paralleled the deterioration and restoration of the capillary bed at the infarct core. It is notable that cerebral blood flow (CBF), as measured by multi‐exposure speckle imaging (MESI) from the brain surface, was restored much earlier than neural activity both at the microinfarct core and at the recording sites (Figure S2a, Supporting Information), consistent with our previous findings in young adult animals.^[^ ^26^ ^]^ However, CBF did not track the vasculature volume fraction at the microinfarct core (Figure S2b, Supporting Information), likely due to the cortical‐depth specific longitudinal response after microinfarcts. Since capillary damage was more pronounced and lasting at deeper cortical regions and speckle imaging of CBF provided a depth‐integrated measurement,^[^ ^47^ ^]^ it is plausible that the detected CBF restoration primarily reflected the reperfusion of shallower layers while being relatively insensitive to finer‐scale vascular disruptions in deeper regions. The agreement between neural activity and capillary volume fraction throughout all measurement periods indicates that the integrity of microvasculature across the cortical depth could serve as a reliable biomarker for neural activity after microinfarct, providing a precise target for assessing post‐microinfarct impact and recovery.

Leveraging depth‐resolved imaging and recording techniques, we revealed a disparity in the cortical depth dependence of neural and vascular deficits following microinfarcts. Neural activity was more severely suppressed at shallow cortical layers, while microvasculature damage was more severe in deeper layers. Several factors may contribute to this disparity. First, although superficial vasculature remained largely intact, transient ischemic insult may have triggered excessive spreading depolarization,^[^ ^48^ ^]^ calcium overload, and metabolic failure in these neurons. Our previous study^[^ ^49^ ^]^ showed that similar targeted photothrombosis induced peri‐infarct depolarizations at the surface, likely exacerbating ionic imbalance and metabolic stress in shallow‐layer neurons. Furthermore, superficial cortical layers rely more on long‐range corticocortical connectivity, which may be disrupted by nearby microinfarcts, leading to prolonged suppression of activity.

We classified recorded neurons into putative FSIs and RS cells and found that following microinfarcts, the excitability of FSIs was severely dampened, whereas the RS neurons were relatively unaffected. The unit‐level changes aligned with network alterations, including disruption of spike‐field coherence in the gamma band, predominantly driven by FSIs, and enhanced population coupling observed exclusively in RS neurons. Our findings that FSIs are especially vulnerable to microinfarcts agree with ex vivo studies using transient ischemia models,^[^ ^50^, ^51^ ^]^ indicating that FSIs, with their higher metabolic demands for sustaining high‐frequency spiking, are more susceptible to ischemic insult than other cell types.^[^ ^52^, ^53^ ^]^

We found a significant reduction in inhibition, contrasting with the excessive inhibition commonly observed in larger‐scale strokes,^[^ ^54^ ^]^ where reducing inhibition has been shown to improve post‐stroke recovery.^[^ ^55^ ^]^ The contrast between those findings and our study highlights a critical distinction in the excitatory/inhibitory (E/I) responses, likely due to the difference in ischemic severity, suggesting that the E/I imbalance varies qualitatively with ischemia scale and severity. While larger strokes induce strong and lasting inhibitory neural responses, microinfarcts may shift the E/I imbalance in the opposite direction, with reduced inhibition and heightened excitation emerging as the dominant responses. Consequently, the therapeutic strategy for reducing ischemic damage and promoting recovery may need to adapt accordingly by prioritizing reducing excitotoxicity and controlling excessive bursting in cases of microinfarcts.

One limitation of the photothrombotic stroke model is its restriction to occluding vessels on or near the brain surface due to the limited penetration depth of light. In this study, we focused on a specific cortical region, the motor cortex. Given the similar anatomical and vascular structures across superficial cortical areas, our findings should apply to microinfarcts in other cortical regions. However, it remains uncertain whether the neuronal and vascular impact in deeper brain regions would be comparable. Additionally, we examined a single isolated microinfarct, which may not account for cumulative effects, and thus may not fully reflect the impact of multiple microinfarcts seen in clinical cases.

## 4 Experimental Section

### Ultraflexible NET Fabrication and Assembly

The procedure of ultraflexible NET fabrication and assembly followed what was described previously.^[^ ^26^ ^]^ Polyimide (PI) (PI2547, HD Microsystems, NJ, USA) was used to construct the insulating layers. Fused silica (soda lime glass 100‐mm DSP (double‐side polished) 550‐µm thick, University Wafer) was used as the substrate instead of silicon to suppress the photovoltaic effect. The thickness of the NET probe was ≈1 µm. The fabrication procedure followed: i) An 80 nm thick nickel sacrificial layer was applied using photolithography and electron beam evaporation, serving as the final release layer. ii) A PI thin film was spin‐coated to achieve a thickness between 450 and 550 nm, followed by a thermal treatment. iii) Electrical trace lines, which connected the bonding pads to individual recording sites, were created using photolithography and electron beam evaporation of Cr/Au, reaching a thickness of 5/120 nm. These trace lines were 1.5 µm in width and 3 µm in pitch. iv) Bonding pads were shaped through photolithography and a layered metal deposition of Cr/Ni/Au, measured at thicknesses of 5/80/20 nm, respectively. v) A second PI layer was applied and thermally processed. An etching mask was then developed using photolithography, and reactive ion etching (RIE) with O_2_ plasma was employed to pattern the vias and define the mechanical structure of the NETs. vi) Individual recording sites in the implanted section were patterned by photolithography and metal deposition using Cr/Au at a thickness of 5/120 nm. NETs used in this study had 32 channels in 4 shanks, 8 recording sites per shank at a spacing of 100 µm center‐to‐center, and a contact size of 30 µm in diameter.

### Animals and Surgery

Ten aged C57BL/6 wild‐type mice (National Institute on aging Aged Rodent Colonies) were used in the experiments. The animals were 16–17 months when NETs were implanted. Mice were anesthetized with 3% isoflurane in an induction chamber and under 1.5–2% anesthesia for NETs implantation surgery. Ethiqa XR (3.25 mg kg^−1^) and dexamethasone (2mg kg^−1^) were administered subcutaneously to reduce pain and inflammation. The craniotomy was performed by removing a 3 mm circular portion of the skull using a dental drill (Ideal Microdrill, 0.4 mm burr, Fine Science Tools, CA, USA) over the motor cortex. Dura mater was removed, and four‐shank 32 channels NETs were implanted perpendicular to the brain surface with 500 µm spacing using tungsten wires as shuttle devices. The backend PCB chip was positioned on the skull part ≈5 mm posterior to the implantation site at a 55‐degree angle. A 3 mm round cover glass (#1, World Precision Instruments, Sarasota, FL, USA) was placed over the craniotomy region. The brain region between the skull and cover glass was sealed with Kwik‐sil adhesive. A burr hole was drilled on the contralateral side of the hemisphere, and a stainless‐steel wire was inserted into the brain as the grounding reference. A layer of vet‐bond tissue adhesive (3M, USA) was applied to cover the exposed skull, and then C&B‐Metabond (Parkell Inc., NY, USA) was applied over the glue to cover the exposed skull as well as stabilize the vertically mounted PCB backend.^[^ ^28^ ^]^ Three animals were excluded from the chronic imaging studies because of cranial window dura regrowth post‐stroke. Four animals were excluded from the chronic electrophysiology studies because of NET channel loss or post‐surgery immune responses. One more mouse was excluded from the depth‐specific electrophysiology analysis due to a lack of precise information on implantation depth. All experiments received approval from the Institutional Animal Care and Use Committee (IACUC) at Rice University and comply with guidelines for the care and use of laboratory animals by the National Institutes of Health. The IACUC approval number is IACUC‐22‐165.

### Targeted Photothrombotic Induction

To induce photothrombotic stroke, animals were lightly anesthetized and head‐fixed on a treadmill under the home‐constructed laser contrast speckle imaging (LSCI) system. Rose Bengal (15 mg mL^−1^, 40 mg kg^−1^) was administered retro‐orbitally and one single penetrating arteriole branch on the brain surface was targeted using 532 nm power laser with an average power density of 3 mW/mm.^2^ The patterned illumination was realized using a ‐ DMD (DLP3010, Texas Instruments), which precisely steered the light to the brain surface based on the location of DMD pixels activated. The selection of the vessel branch for inducing photothrombosis was based on flow direction data acquired from 2P line‐scanning of both penetrating and ascending vessel branches. The selected vessel segment was subsequently transformed into DMD coordinate space through an affine image transformation. The illumination pattern was created using FOIL Software (Dynamic Light Inc., Austin, TX) to conform to the contours of the selected vessel branch closely. CBF during stroke induction was monitored using LSCI (Figure S3a, Supporting Information) with a 685‐nm laser diode (50 mW; HL6750MG, Thorlabs) to track local ischemic progression (Figure S3b, Supporting Information). All animals were closely monitored for weight loss and any other signs of discomfort after the induction of photothrombosis.

### Longitudinal CBF Quantification

MESI was utilized to measure longitudinal CBF change post‐microinfarct. The system used a volume holographic grating wavelength‐stabilized, single frequency 785 nm laser diode (LD785‐SEV300, Thorlabs) to illuminate the cranial window and triggering 15 exposures on a camera (acA1920‐155 um; 1920 × 1200 pixels, Basler AG) with durations varying from 0.05 to 80 ms. Laser intensity was adjusted to maintain a consistent total light output across different camera exposures to mitigate the impact of shot noise. An objective lens (AF‐S NIKKOR 50 mm F/1.8G, Nikon), capturing a field of view of 2.92 mm by 4.02 mm, collected the backscattered light and projected it onto the camera. Three to four measurement sessions were conducted to establish BL, and subsequent post‐microinfarct MESI measurements were normalized to the average of these BL values. MESI imaging sessions for all mice were performed when mice were awake and head‐fixed on a customized treadmill.

### 2P Imaging Acquisition and Processing

2P imaging was conducted using a multiphoton laser scanning microscope with a 16 × water immersion objective (Nikon), and laser power was tuned to ≈50 to 250 mW at 930 nm wavelength. Blood serum was labeled with FITC Dextran (2M Da). Stacks were taken at 2.63 s per frame in a 1.1 mm × 1.1 mm field of view. Imaging sessions with severe shadowing effects from considerable vessel shadowing and dura regrowth were excluded from the imaging analysis.

The 2P imaging processing pipeline was as described previously.^[^ ^26^ ^]^ To preprocess the 2P data, raw data were smoothed by a 3d Gaussian filter with 1 µm full width at half maximum (FWHM) kernel size for shot noise removal. The background was subtracted using a rolling ball algorithm (50 µm diameter). To correct the inhomogeneous intensity caused by light scattering during tissue penetration, the intensity of each z‐stack slice was normalized to the grand mean intensity of slices across all imaging sessions for this animal. The non‐homogeneous intensity of the x‐y plane resulting from brain curvature, shadowing from NETs, or large surface vessels was corrected using a linear combination of three Gaussian filters with kernel sizes of 8, 14, and 30 µm, respectively. The kernel sizes correspond to the diameter of three types of brain vessels. After preprocessing, Imaris (Bitplane, Belfast, United Kingdom) was used to generate binary imaging stacks using the local contrast thresholding method. To correct the discrepancy between this binarization method and volume fraction estimation using FWHM, the vessel diameter ratio between raw and binarized imaging stack was calculated and averaged to generate a scaling factor for correction. The region of interest to measure vasculature volume fraction for longitudinal comparison was selected based on the session post microinfarct induction when the vascular occlusion was most severe on x‐y maximum intensity projection image at 200–300 µm below the brain surface. In Figure 2b, volume fraction values were normalized to BL values for each animal and then averaged across animals. To compare vasculature recovery at different cortical depths, normalized vasculature volume fraction values were calculated at increments of 100 µm, up to a depth of 500 µm below the brain surface. Vasculature deficits at each cortical depth (Figure 3c) were quantified by computing the integral of these normalized volume fraction curves over time.

Capillary stalls were defined by the criteria that immobilized RBCs cast a shadow on the vessel segment for >20 s from the raw 2P imaging z‐stack. Stalls were manually identified and quantified using Fiji (Open source, ImageJ.net) by examining the Z‐stack through imaging in a forward and reverse manner along the z‐axis direction to ensure accuracy.

### Capillary Blood Flow Data Acquisition and Analysis

Animals were administered FITC‐dextran (0.1 ml, 5% w/v, Sigma‐Aldrich) through i.v. injection to label the serum. A distance of ≈30 µm along capillaries both adjacent to and far away from microinfarct sites was scanned 256 times, which we refer to as one frame, at 1 to 2 ms per scan, along its central axis to obtain strip‐shape images for estimation of velocity and flux of red blood cell (RBC), and hematocrit (HCT). 5–10 frames were averaged for each capillary to obtain the final values. The method to quantify the RBC velocity, flux, and hematocrit values from 2P line scans is as described previously.^[^ ^26^ ^]^ An open‐source MATLAB script^[^ ^56^ ^]^ was used to calculate the average RBC velocity for each capillary. Another customized MATLAB script counted the RBC streaks to measure RBC flux within the same time frames. To determine hematocrit (HCT) values over time, frames were preprocessed and binarized, and the proportion of dark stripes—indicative of red blood cells—was calculated for each capillary. The accuracy of the automated scripts for quantifying RBC velocity, flux, and hematocrit was confirmed by manually analyzing a subset of the data.

### Electrophysiology Data Acquisition and Analysis

The extracellular recording was taken using IntanRHD 2000 system sampling at 30 kHz with stainless steel in the contralateral hemisphere as grounding reference. The impedance of all contacts was measured before each session, and contacts with an impedance of >2 MΩ were excluded from the electrophysiology measurement. Mice were head‐restrained and awake under a custom treadmill during the acquisition of all electrophysiology data. Each measurement session lasted 30 min. The LFP power was computed by band‐pass filtering at 0.5–6000 Hz and median subtracted. LFP power within a 1 s non‐overlap window was computed using the Fourier transform. The mean LFP power within the 30 s was average to represent the average LFP power of each session. Spike sorting was carried out using MountainSort 4^[^ ^57^ ^]^ after raw data was highpass filtered at 300 Hz. The spike detection threshold was set to 4.5 SD, and an adjacency radius of 100 was used. Waveforms of sorted units were then manually curated to reject noise clusters and correct over‐split units. Axon‐like waveforms were also removed to reduce contamination of layer‐specific analysis. LFP and spike firing rate were normalized against mean values of BL and then averaged across animals in Figure 2f,g. The normalized average firing rates across cortical layers (Figure 3d) were computed by averaging across animals the firing rates of single units at electrode contact sites, which were assigned to specific cortical layers based on contact locations. The mean values were then normalized to the mean values of BL levels at each cortical layer. The locations of the sorted units, as in Figures 1e and 4a, were estimated as the weighted averages of the locations, with weights corresponding to the spike amplitudes detected on channels within the same shank.

To compute spike‐LFP phase locking, raw data sampled 30k Hz was downsampled to 10k Hz, and filtered within bands of 2 Hz using a Kaiser Window FIR filter with 60 dB attenuation in stop bands, 0.01 dB pass‐band ripple, and transition bands of 1 Hz. Hilbert transform was applied within the 2 Hz narrow band signals to extract phase and magnitude information (Figure S4c, Supporting Information). The Kappa parameter from the Von Mises distribution was obtained from the spike phase polar histogram of the functional electrode contact site.^[^ ^58^ ^]^ Neurons were considered strongly phase‐locked and included in the analysis if their Kappa values from spike‐phase polar histogram exceeded the 95% threshold of a shuffled distribution.

Population rate (Figure 5d) was calculated by summing all detected spikes within a 1 ms bin and smoothed using a Gaussian filter with a half‐width of 12 ms. Population coupling strength^[^ ^59^ ^]^ of single unit *x* was computed as:

(1) $$c_{x}=\frac{1}{\left|f_{x}\right|}\int f_{x}\left(t\right)\sum_{x\neq y}\left(f_{y}\left(t\right)-\mu_{y}\right)dt$$

Here, f represents the smoothed firing rate of a single unit, μ is its mean firing rate, and |f| denotes the normalization factor, which is the total number of spikes. To compare population coupling strength across sessions, values were normalized by the median of shuffled population coupling strength. Spike shuffling was performed by first dividing spike trains into non‐overlapping 1 ms bins, and therefore, a binary matrix was created where each column represents a time bin and each row represents a single or a multi‐unit from the same shank. Then, the constructed matrix was binarized where a “1” represents the time points spiking activity occurred and a “0” represents no spiking activity occurred. Shuffling was then performed by randomly selecting 2‐by‐2 submatrices and swapping elements to maintain row and column sums. Multiple iterations produced a pool of shuffled population coupling coefficients and the median value used for normalization.

### Tissue Processing and Lesion Volume Analysis

Animals were anesthetized with 3.0% isoflurane and transcranial perfused with 50 ml 0.1 m phosphate‐buffered saline and 50 ml 4% paraformaldehyde (PFA). Perfused brains were postfixed in 4% PFA for 24 h and cryoprotected in 30% sucrose phosphate‐buffered saline (PBS) solution for 72 h. The brain was extracted by first snap‐frozen within a tube in a liquid nitrogen bath to allow brain skull separation and, second, carefully dissected with NETs embedded. Extracted brains were again cryoprotected in 30% sucrose PBS solution until the tissue sank. The cryoprotected tissue was snap‐frozen in an isopentane‐liquid nitrogen bath and sectioned into 30 µm thick coronal slices (Leica CM1950). Every section near the microinfarct region was mounted on the slides (Fisherbrand Superfrost Plus Microscope Slides) to avoid missing the microinfarct region. Mounted slides were air‐dried overnight and Nissl‐stained with toluidine blue (Millipore Sigma, toluidine: sodium borated: dH_2_O in weight equal to 1:2:800) for microinfarct identification and volume reconstruction. The images were analyzed in Fiji (Open source, ImageJ.net), and the microinfarct lesion was identified as the region with significantly lower Nissl body density than the surrounding healthy tissue.

### Statistics

Data are presented as mean ± SE. The data from Figure 2c,d (left, right), 2i, and Figure 5b (top), 5d, and Figure S1a,d–i and S4a,b (Supporting Information) were statistically evaluated using one‐way ANOVA with Tukey's post‐hoc test for multiple comparisons. The data from Figure 2d (middle), Figure 4d,e (right), 4f (right), and Figure 5b (bottom) were statistically evaluated using Kruskal‐Wallis with Dunn's post‐hoc test for multiple comparisons. The data from Figure 3f were evaluated using the Mann‐Whitney U test. A p‐value of 0.05 or lower was considered statistically significant. The assumption of normality and equal variance was considered if the sample size within each group was <30. If the sample size exceeded 30, only the equal variance assumption was considered, as one‐way ANOVA is robust to non‐normality data in large samples. All statistical tests were performed using MATLAB and OriginPro.

## Conflict of Interest

The authors declare no conflict of interest.

## Author Contributions

The conceptualization of the project was carried out by L.L. and Y.J. The methodology was developed by Y.J., F.H., H.R., Y.S., J.Z., X.L., R.Y., and H.Z. The software development involved Y.J., F.H., H.R., Y.S., J.Z., X.L., and H.Z. The investigation was conducted by Y.J., F.H., H.R., R.Y., and X.L. The analysis was led by Y.J., and the visualization work was done by Y.J. and L.L. The writing was a collaborative effort between L.L. and Y.J. Supervision was provided by L.L. and C.X., while L.L. also handled project administration. Finally, the funding acquisition was managed by L.L. and C.X.

## Supporting information



Supporting Information

## Data Availability

The data that support the findings of this study are available from the corresponding author upon reasonable request.
