# Supplementary material for: Longitudinal, Multimodal Tracking Reveals Lasting Neurovascular Impact of Individual Microinfarcts
Source: Adv Sci (Weinh). 2025 Mar 31;12(22):2417003. doi: 10.1002/advs.202417003 (PMC12165101; doi:10.1002/advs.202417003)
Supplement: Supplementary file 1 — Supporting Information [file ADVS-12-2417003-s001.docx]

**
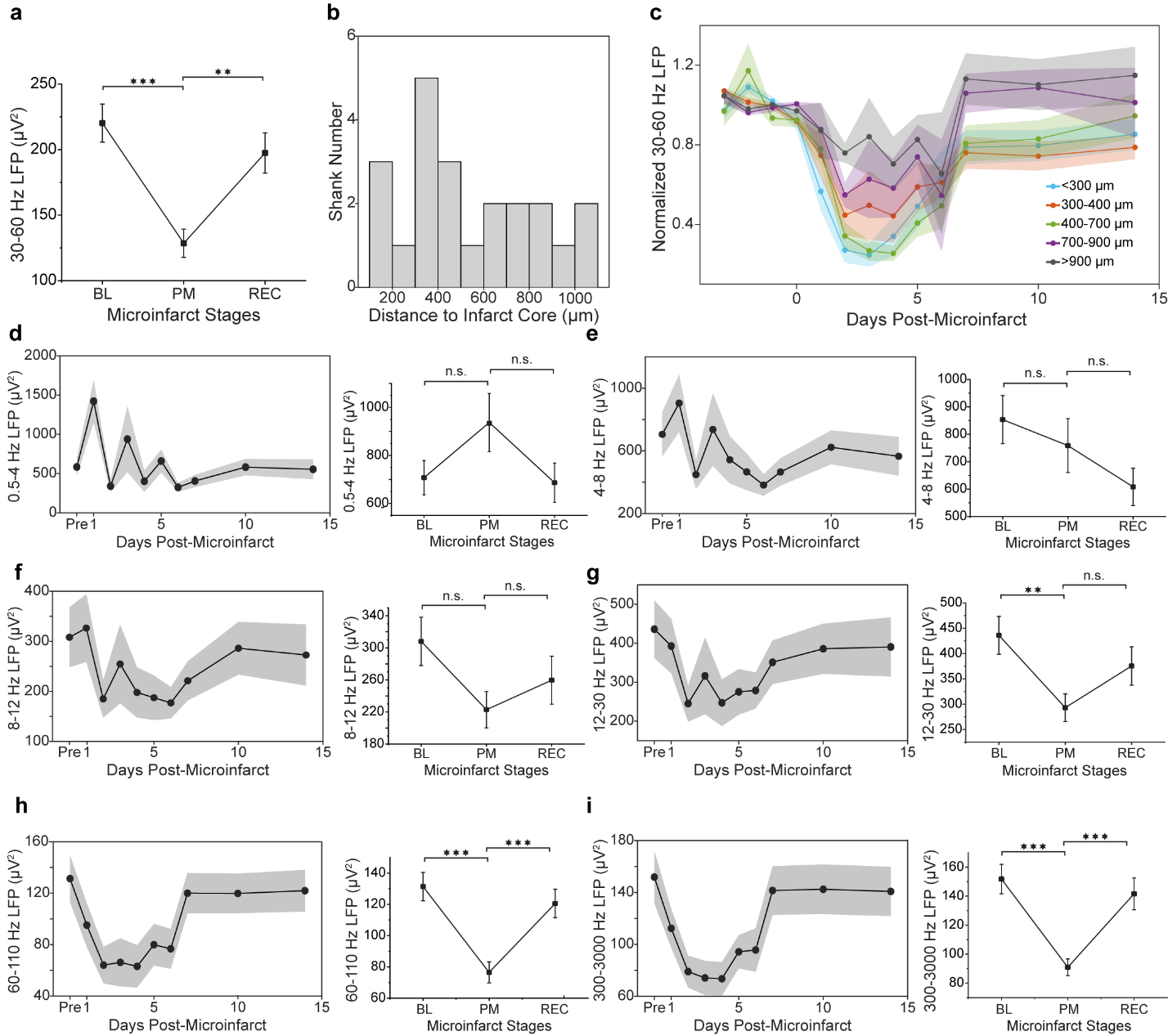
Figure S1.** Distance-frequency-dependent LFP recovery post microinfarct. a) LFP spectral power of 30-60 Hz across three stages of the microinfarct. b) Distance to infarct core distribution for all 22 shanks. c) Average normalized LFP spectral power at 30-60 Hz at various distance ranges from the microinfarct core. Shade indicates ± SE (n = 22 shanks). d-i) Average spectral power of LFP (left, n = 22 shanks) at all measurement sessions and statistical analysis of spectral power across three stages of the microinfarct (right) at 0.5-4 Hz (d), 4-8 Hz (e), 8-12 Hz (f), 12-30 Hz (g), 60-110 Hz (h), 300-3000 Hz (i). Significance levels: n.s., no significance; **^🟋^***P*<0.05; **^🟋🟋^***P*<0.01; **^🟋🟋🟋^***P*<0.001.

**
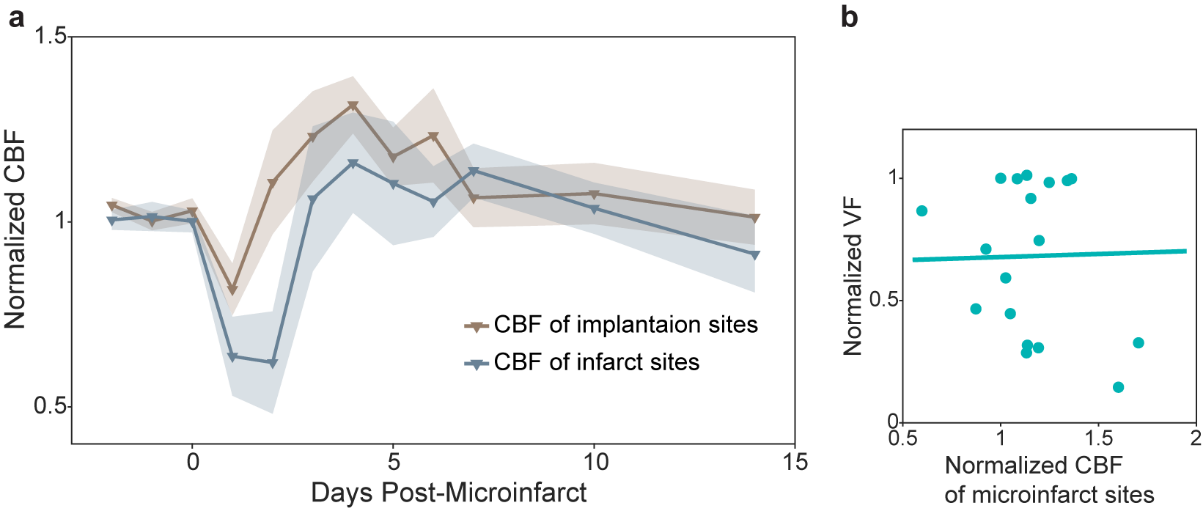
**

**Figure S2.** Uncoupling of neural activities and cortical surface CBF post microinfarct. a) Normalized cortical surface CBF measured by MESI as a function of day relative to microinfarct at implantation and infarct core sites. Shade indicates ±SE (n=6 mice). b) Scatter plots of normalized CBF at microinfarct sites vs. normalized capillary volume fraction. Pearson’s correlation coefficient: ρ=-0.026, P=0.9.


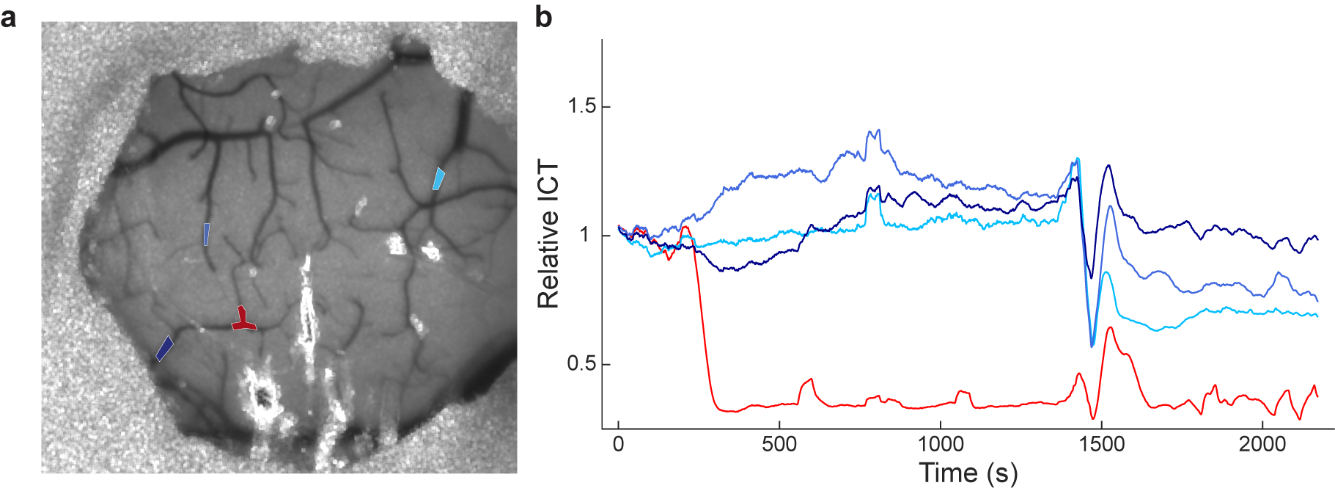
**Figure S3.** Acute LSCI imaging following stroke induction. a**)** Representative speckle images illustrating vessel branches to measure CBF during stroke induction session (occluded vessel branch labeled in red). b) Relative CBF change within ROIs labeled in a).

**
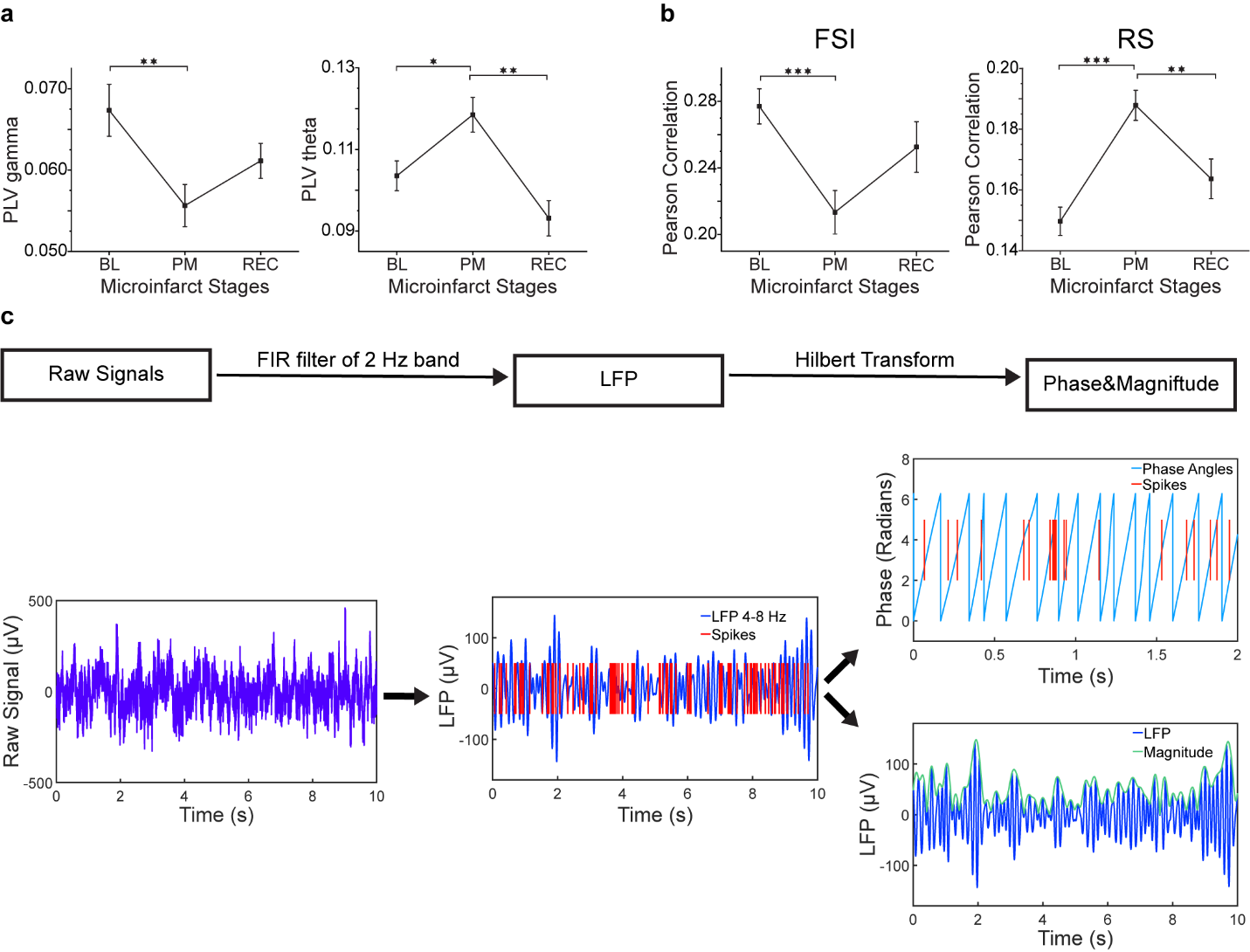
Figure S4.** Spike-field phase locking and population coupling change post microinfarct. a) Average phase locking values (PLV) of gamma band (left) and theta band (right) for all channels that passed permutation testing from 4 mice at three stages following the microinfarcts. Error bars represent SE. b) Average Pearson correlation values of individual unit activities and population spiking activities for all units from 6 mice at three stages following the microinfarcts. Error bars represent SE. Significance levels: n.s., no significance; **^🟋^***P*<0.05; **^🟋🟋^***P*<0.01; **^🟋🟋🟋^***P*<0.001. c) Signal processing flow chart for phase and magnitude extractions at the frequency band of interest (top flow chart). Example raw trace and resulting theta-band LFP, spike-LFP phases, and magnitude envelop (bottom flow chart).
